# Prospective associations between stressful life course events and clusters of lifestyle behaviours

**DOI:** 10.1186/s12889-025-24110-3

**Published:** 2025-09-24

**Authors:** Raymond Vooi Khong Siew, Susan J. Torres, Charlotte Juul Nilsson, Gavin Abbott, Åse Marie Hansen, Nicolas Cherbuin, Liana Leach, Peter Butterworth, Anne I. Turner

**Affiliations:** 1 School of Health and Social Development, Institute for Health Transformation, Faculty of Health, Deakin University, Geelong, Australia; 2 Section of Social Medicine, Department of Public Health, University of Copenhagen, Copenhagen, Denmark; 3 Institute for Physical Activity and Nutrition, School of Exercise and Nutrition Sciences, Deakin University, Geelong, VIC 3220 Australia; 4 National Research Centre for the Working Environment, Copenhagen, Denmark; 5 National Centre for Epidemiology and Population Health (NCEPH), The Australian National University, Canberra, Australia; 6 Centre for Social and Early Emotional Development, School of Psychology, Deakin University, Melbourne, Australia

**Keywords:** Life stress, Childhood adversity, Trauma, Chronic stress, Lifestyle behaviours

## Abstract

**Background:**

The adoption of health-damaging lifestyle behaviours may be a coping mechanism stemming from maladaptive behavioural stress response to stress exposure, but little is known about the influence of stressors on health-damaging lifestyle behaviour clusters. This study aimed to identify clusters of lifestyle behaviours based on six lifestyle-related behaviours and to examine the associations between childhood adversity, stressful and traumatic life events, and the clusters of lifestyle behaviours in community-dwelling Australian adults.

**Methods:**

This prospective study comprises 1773 adults aged 40–44 years at baseline from the Personality and Total Health (PATH) Through Life Study. Data on childhood adversity, stressful life events, traumatic life events were obtained from Wave 1 in 2000/2001 to Wave 3 in 2008/2009 and lifestyle behaviours were obtained at Wave 4 in 2012/2013 using self-reported surveys. Latent class analysis was conducted to identify distinct clusters of lifestyle behaviours using data from physical activity, alcohol consumption, insomnia, fruit and vegetable intake, and smoking status. Multinomial logistic regression analysis examined the associations between stressors and lifestyle behaviour clusters.

**Results:**

Three clusters of lifestyle behaviours labelled ‘Poor sleep quality, low vegetable intake’ (58.8%), ‘Inactive, risky alcohol use, smoker’ (3.3%), and ‘Inactive, low fruit and vegetable intake, poor sleep quality, smoker’ (35.9%) were identified. Exposure to stressful life events was associated with higher likelihood of belonging to the ‘inactive, low fruit and vegetable intakes, poor sleep quality, smoker’ cluster as opposed to the ‘poor sleep quality, low vegetable intake’s cluster (RRR = 1.42, 95% CI=[1.12, 1.81]) after adjusting for age, sex, educational level and income.

**Conclusions:**

Our findings revealed an association between exposure to stressful life events and clustering of being physically inactive, low fruit and vegetable intake, poor sleep quality and smoking. Consequently, implementing health promotion strategies to target multiple lifestyle behaviours may be beneficial in reducing the cumulative impact of stress on health.

**Supplementary Information:**

The online version contains supplementary material available at 10.1186/s12889-025-24110-3.

## Background

Stress is a risk factor for physical and mental health disorders and poses a concern for public health [1]. Based on the 2021 National Mental Health and Wellbeing survey, 15% of Australian adults reported that they experienced clinically significant levels of psychological distress [2]. Psychological distress can arise from negative life experiences including stressful life events, traumatic life experience, illness, financial problem, and family relationships [3]. Furthermore, childhood adversity contributes to the development of mental health disorders [4]. Addressing these chronic illnesses comes at a significant economic cost, adding strain to the health system [5].

Chronic exposure to stressors has been proposed to be an antecedent to chronic illnesses such as cardiometabolic diseases [1, 6]. Chronic overactivation of the hypothalamic-pituitary-adrenal (HPA) axis leads to sustained elevation of glucocorticoids, namely cortisol. Elevated cortisol increases blood pressure via sympathetic activity, increases systemic inflammation via elevated pro-inflammatory cytokines and reduces insulin sensitivity through altered glucose metabolism. These pathophysiological processes result in dysregulation of neuroendocrine, metabolic and immune systems, known as allostatic load [6].

These pathophysiological processes are compounded by stress-induced behavioural adaptations. Chronic stress can increase individuals’ susceptibility to maladaptive lifestyle risk factors contributing to metabolic stress, weight gain and insulin resistance, thereby synergistically exacerbate both biological and behavioural risk profiles [7]. For example, stress acts in the brain reward system to increase appetite for highly-palatable food high in sugar and fat [8]. Stress can also adversely affect sleep quality, leading to sleep deprivation [9]. Stressed individuals may also neglect exercise as they are preoccupied with extant stressors [10]. Additionally, some research has suggested that stressed individuals may use alcohol and smoking as an avoidance-focused coping mechanism against stressors [11, 12]. The addictive nature of alcohol and smoking can subsequently facilitate a harmful cascading process that reduces life expectancy [13]. Taken together, these findings underscore the importance of addressing stress not only as a psychosocial factor but also as a biological catalyst for chronic disease [14]. To reduce the burden of diseases attributable to stress, there is a need to identify and act on these modifiable risk factors that could prevent the early onset of diseases.

Lifestyle behaviours in response to stress do not occur in isolation, instead, they co-occur to form clusters [15]. These clusters of unhealthy lifestyle behaviours can further accelerate the progression to early onset of lifestyle-related diseases, leading to increased mortality rates [16]. A longitudinal study of 1080 adults in the United Kingdom found that the combination of health-damaging lifestyle behaviours was associated with increased mortality rates [17]. Examining the impact of common stressful events across the life course about the clustering of adverse lifestyle behaviours is important because it provides a more holistic picture of the intersection between stress and the mechanisms that drive chronic conditions.

Previous research investigating stress and lifestyle behaviours has examined the perception of stress and psychological distress more broadly rather than focusing on more specific stressors. A small number of studies have investigated the link between stress and lifestyle behaviours. In a large cross-sectional study (*n* = 14,868) in Denmark, individuals with low socioeconomic position and higher perceived stress were at a higher risk of presenting with co-occurrence of multiple health-damaging lifestyle behaviours including low fruit and vegetable intakes, smoking and physical inactivity [18]. Another cross-sectional study also found that university students who experienced higher level of psychological distress were more likely to report various combinations of co-occurring unhealthy lifestyle behaviours such as low vegetable intake, consuming soft drink and energy drinks, physical inactivity, drug and alcohol use and smoking [19]. The study addresses a critical knowledge gap by investigating the longitudinal associations between exposure to specific stressors and the clustering of lifestyle behaviours to explore temporality within adulthood using a prospective study design with information collected from four waves of data collection. The findings from this study have the potential to guide early public health interventions, targeting high-risk individuals based on their stress exposure history. We hypothesised that exposure to stressors would be associated with clustering of unhealthy lifestyle behaviours. This study aimed to identify clusters of lifestyle behaviours and the association with childhood adversity, stressful and traumatic life events in community-dwelling middle-aged Australian adults.

## Methods

### Participants

**Fig. 1:**
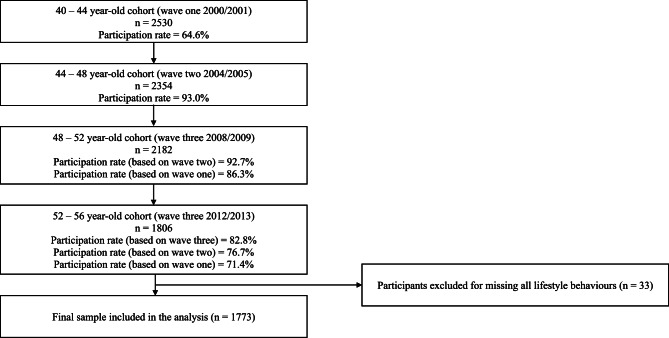
Study profile

Participants were from the PATH study, a prospective study jointly hosted by the Australian National University (ANU) and the University of New South Wales (UNSW) Australia designed to investigate individual health and well-being trajectories across the life course. A full description of the study has been published elsewhere [20]. Participants who resided in the Australian Capital Territory and the neighbouring town of Queanbeyan were randomly selected from the Australian Electoral Rolls and invited to participate in the study. Using the 2530 middle-aged cohort participants (40–44 years) who participated in the data collection at Wave 1 in 2000/2001, 1806 (71.4%) participants remained in the study at the Wave 4 follow-up data collection in 2012/2013. The analytic sample included 1,773 participants who completed all four waves of data collection without missing information on all lifestyle behaviours (Fig. 1). At each wave, participants provided written informed consent and the study protocol was approved by the Human Research Ethics Committee of the Australian National University (HREC 2010_542). An ethics exemption was obtained from the Deakin University Human Research Ethics Committee for the use of de-identified data in this manuscript (2021-016).

### Study design

The analysis draws upon data on sociodemographic characteristics (age, sex, education level and personal income), measures of childhood adversity, stressful life events, traumatic life events collected between Wave 1 and Wave 3, and lifestyle behaviours collected at Wave 4. Data on childhood adversity was measured at Wave 1. Data on recent experience of traumatic life events were measured at Wave 2 and Wave 3. Data on stressful life events was measured at Wave 1, Wave 2, and Wave 3. The outcome measures for the current study are lifestyle behaviours (physical activity, alcohol consumption, fruit and vegetable intake, insomnia, and smoking status) assessed at Wave 4 at the 12-year follow-up. The full survey questionnaire can be found in Supplementary file 1.

### Stressors exposure

#### Childhood adversity

Childhood adversity was measured at Wave 1 using 13 items and participants were asked if they had not had experienced childhood happiness; parents did not do their best for them; neglect; strict upbringing; poverty; verbal abuse by a parent; humiliation; witnessed physical or sexual abuse of another family member; physical abuse of others in family; physical punishment; sexual abuse; normal upbringing; other mistreatment not specified during childhood [21]. Participants were categorised as ‘yes’ if they had experienced one or more of the events at Wave 1 or ‘no’ if they did not experience childhood adversity at all.

#### Stressful life events

Stressful life events in the last six months were measured at Wave 1, Wave 2, and Wave 3 using 13 items with participants asked if they had experienced the following stressful life events relating to personal illness, injury, and assault; close family member serious illness, injury, and assault; parent, child or partner bereavement; close family friend or relative bereavement; relationship breakdown; serious problem with close friend, neighbour or relative; crisis or serious disappointment in work; at risk of job loss; unemployment; fired from work; financial crisis; trouble with the legal system; and theft [22]. There were two response options “yes” and “no”. Stressful life events were dichotomised into ‘yes’ if they have ever experienced stressful life events from Wave 1 to Wave 3 or ‘no’ if they did not experience stressful life events at all.

#### Traumatic life events

Traumatic life events were measured at Wave 2 and Wave 3 using 10 items and participants were asked if they had ever experienced trauma from combat war experience; life threatening accident natural disasters; witness a serious injury or murder; sexual assault or harassment; physical assault; threatened with a weapon; held captive; or kidnapped; torture or victim of terrorists; other upsetting events [23]. Traumatic life events were dichotomised into ‘yes’ – if they have ever experienced traumatic life events or ‘no’ if they did not experience traumatic life events at all.

### Lifestyle behaviours

#### Physical activity

Physical activity was determined using the Active Australia Survey and included the total time spent walking, and performing moderate and vigorous activity in the previous week [24]. Total time spent on physical activity per week was calculated using the formula: light walking (minutes) + moderate activity (minutes) + vigorous activity x 2 (minutes). Participants were categorised according to the national recommendation [25]: did not meet guideline (< 150 min activity/week) or met guideline (> 150 min activity/week).

#### Insomnia

Insomnia or sleep problems were determined by asking if participants have insomnia or sleep problems with responses as ‘yes’ or ‘no’. Participants were also considered as having insomnia or sleep problems if they reported taking sleep medications.

#### Alcohol consumption

Alcohol consumption risk assessment was determined using the Alcohol Use Disorders Identification Test (AUDIT) [26]. The AUDIT is a brief screening tool to identify the risk of excessive drinking pattern based on participants’ responses to the 10 items. The AUDIT produces a total score ranging from 0 to 40, and following the AUDIT guidelines participants were categorised based on their risk of excessive alcohol consumption as either abstinence/low risk (0–7) or at risk (8-40) [26].

#### Fruit and vegetable intake

Fruit and vegetable consumption based on two questions in which participants reported their daily consumption in the following categories: 1 serve or less; 2–3 serves; 4–5 serves; 6 serves or more; do not eat fruit/vegetables. Participants were categorised according to their adherence to the recommended intakes from the Australian Guide to Healthy Eating [27]. Therefore, participants either had a high intake of fruit (2 serves or more per day) or a low intake (less than 2 serves per day). Similarly, participants either had a high intake of vegetables (4 serves or more per day) or a low intake (less than 4 serves per day).

#### Smoking

Smoking status was determined by asking if they were a current smoker and categorised as: current-smoker or non-smoker.

### Potential confounding variables

Age, sex of participants and highest education were self-reported at baseline. Age was recorded as a continuous variable whereas participants’ sex was binary (male/female). Participants were asked the highest level of education and their responses were grouped into the following categories: school (primary and secondary), diploma (certificate/diploma/apprenticeship), and tertiary education (bachelor’s degree and higher).

### Statistical analysis

The latent class analysis was conducted without the inclusion of auxiliary variables to avoid unintentional influence on class formation according to Nylund-Gibson and Choi [28]. Latent classes were enumerated using six dichotomised lifestyle behaviours (physical activity, alcohol consumption risk, fruit and vegetable intake, insomnia, and smoking status) to identify clusters (latent classes) of lifestyle behaviours. We began with fitting a model with one class with one additional class in the subsequent model until the ‘optimal’ solution was found [29]. We selected the ‘optimal’ solution based on interpretability and the following model fit statistics: Akaike Information Criteria (AIC), Bayesian Information Criteria (BIC), entropy values, Lo-Mendell-Rubin Likelihood Ratio Test (LMR-LRT), and Bootstrap Likelihood Ratio Test (BLRT). Smaller AIC and BIC values indicate better model fit whereas a higher entropy value indicate participants are more accurately classified into the correct classes. LMR-LRT and BLRT compare *k* vs. *k-1* class models, where a significant p-value (*p* < 0.05) indicated *k* class is better than the *k-1* model [29]. Models were estimated using full information maximum likelihood as a mean of handling the missing data.

**Fig. 2:**
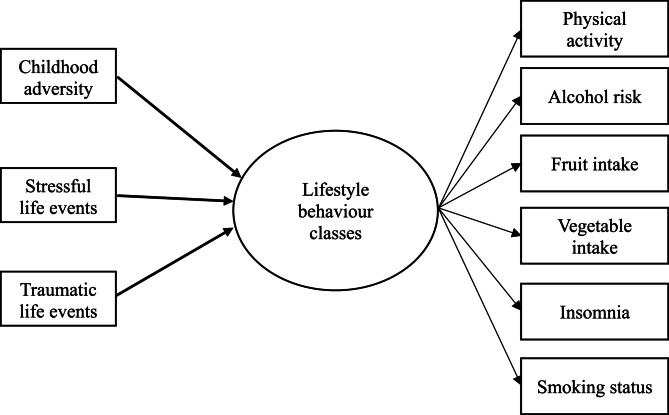
Conceptual model for the associations between childhood adversity, stressful life events, and traumatic life events on latent classes derived from lifestyle behaviours

Multinomial logistic regression was used to examine how the exposures to childhood adversity, stressful life events, or traumatic life events were associated with latent classes of lifestyle behaviour classes (Fig. 2). Model 1 examined the association between stressors and clustering of lifestyle behaviours and adjusted for age, sex. Model 2 examined the association between stressors and clustering of lifestyle behaviours adjusted for educational background and personal income level. Multivariate Imputation by Chained Equations were conducted to handle the missing values in the exposure variables. Thirty imputations were generated, the estimates pooled, and the average point estimates reported.

We used one-way ANOVA to test differences in age between latent classes. Pearson’s chi-square tests were used to identify differences in categorical sociodemographic characteristics among latent classes. We also examined differences in sociodemographic characteristics, between our analytic sample and those lost to follow-up. Statistical analyses were performed using Stata SE 17.0 and Mplus 8.7 (Muthen & Muthen, Los Angeles, CA, USA). Statistical significance was set at *p* < 0.05.

## Results

### Latent classes

The goodness-of-fit statistics supported a three-class model as the most parsimonious model for our analytic sample. The lowest BIC supported a two-class model, while AIC supported a four-class model; a three-class model is the ‘middle ground’ between the two statistics. Furthermore, the LMR-LRT and BLRT results also supported a three-class model (Table 1).

**Table 1:** Latent class analysis goodness-of-fit statistics for one- to four-class models (*n* = 1773)

Model	Log-likelihood	df	AIC	BIC	Entropy	LMR-LRT *p*-value	BLRT *p*-value	Smallest class (%)
1-class	−5342.03	6	10696.06	10728.95	-	-	-	-
2-class	−5250.41	13	10526.82	10598.82	0.46	< 0.001	< 0.001	38.4
**3-class**	**−5237.10**	**20**	**10514.20**	**10623.81**	**0.60**	**0.02**	**< 0.001**	**3.3**
4-class	−5227.79	27	10509.57	10657.54	0.68	0.08	0.06	2.7

Bolded is the class solution selected as the best fitting

*df* degree of freedom for the model, *AIC* Akaike Information Criteria, *BIC* Bayesian Information Criteria, *LMR-LRT* Lo-Mendell Ruben likelihood ratio test, *BLRT* Bootstrap likelihood ratio test

**Fig. 3:**
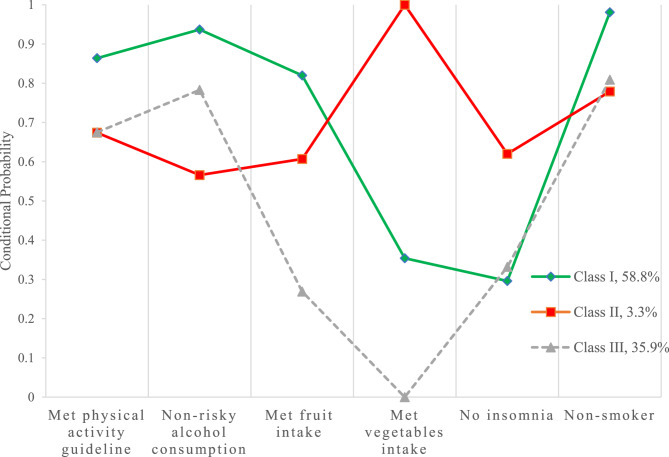
Conditional probabilities of lifestyle behaviours by latent class membership. Class I represents “Poor sleep quality, low vegetable intake ” cluster, Class II represents “Inactive, risky alcohol use, smoker” cluster, Class III represents “Inactive, low fruit and vegetable intake, poor sleep quality, smoker” cluster

Figure 3 shows the conditional probabilities of lifestyle behaviours for each of the three latent classes. Most participants (58.8%) were classified as having the least number of behaviour risk factors – labelled “poor sleep quality, low vegetable intake” (Class I). They had highest probabilities of meeting daily physical activity guideline (> 150 min/week) and the recommended fruit (2 or more serves/day), with moderate probabilities of meeting vegetable (4 or more serves/day) intakes and insomnia, and the lowest probabilities of alcohol misuse (AUDIT score < 8) and being a current smoker. The smallest number of participants (3.3%) were labelled “inactive, risky alcohol use, smoker” (Class II). They had a lower probability of meeting the physical activity guidelines, and a higher probability of being risky alcohol drinkers, and smokers than the participants in Class I. A relatively larger number of participants (35.9%) were labelled “inactive, low fruit and vegetable intake, poor sleep quality, smoker” (Class III). They had a lower probability of meeting the physical activity guidelines, consuming adequate fruit and vegetable intake and they had a higher probability of poor sleep quality and being a smoker than participants in Class I.

### Demographic characteristics

In our study sample of 1773 adults, 54% were females and age ranged between 40 and 44 years at baseline. Participants in the Class I had a higher proportion of participants with tertiary education than other clusters (Table 2). There was also a higher proportion of female than male in Class II while Class I and Class III had equal distributions (Table 2).

**Table 2:** Sociodemographic characteristics of participants in latent classes by clusters of lifestyle behaviours, class I represents “poor sleep quality, low vegetable intake” cluster, class II represents “inactive, risky alcohol use, smoker” cluster, class III represents “inactive, low fruit and vegetable intake, poor sleep quality, smoker” cluster

	Class I (*n* = 1042)	Class II (*n* = 58)	Class III (*n* = 673)	*p*-value
Age, mean (sd)	42.7 (1.5)^a^	42.7 (1.4)^a^	42.5 (1.5)^b^	< 0.01
Sex (%)				
Male	427 (41.0)	15 (25.9)	374 (55.6)	< 0.01
Female	615 (59.0)	43 (74.1)	299 (44.4)	
Educational background (%)				
School	216 (20.7)	14 (24.1)	188 (27.9)	< 0.01
Diploma	324 (31.1)	19 (32.8)	261 (38.8)	
Tertiary education	502 (48.2)	25 (43.1)	224 (33.3)	
Household income, per week (%)				0.56
Less than 300	16	1	15	
300 – less than 575	41	4	32	
575 – less than 1075	119	4	83	
1075 – less than 1700	186	16	138	
1700 – less than 2400	209	11	139	
More than 2400	407	21	240	
Stressful life events (%)				
Yes	286 (27.5)	24 (41.4)	237 (35.2)	< 0.01
No	619 (59.4)	25 (43.1)	340 (50.5)	
Childhood adversity (%)				
Yes	629 (60.4)	40 (69.0)	422 (62.7)	0.25
No	410 (39.4)	17 (29.3)	248 (36.8)	
Traumatic life events (%)				
Yes	333 (32.0)	27 (46.6)	242 (36.0)	0.03
No	709 (68.0)	31 (53.4)	431 (64.0)	

Different superscript letters indicate differences between classes tested using one-way ANOVA for continuous variable and Pearson’s chi square test for categorical variables

Compared with participants who were lost to follow-up at Wave 4, there was a slightly lower proportion of participants who had completed tertiary education and diploma in our sample than those who were lost to follow-up (33.2% and 42.4% respectively) (data not shown).

### Associations between latent classes and stressors

Multinomial logistic regression analysis estimated that the relative risk ratio of being in Class III were 1.4 times higher for participants who had experienced cumulative stressful life events (RRR = 1.42, 95% CI = [1.12, 1.81], *p* < 0.01) compared to the referent cluster (Class I) (Table 3). Although the p-value is not significant at the 95% level, the estimated relative risk of being in Class II were 1.8 times higher for participants who have experienced stressful life events (RRR = 1.81, 95% CI= [1.00, 3.30], *p* = 0.05) compared to the referent cluster of Class I. However, there were no significant associations between childhood adversity and traumatic life events with the latent classes (Table 3).

**Table 3:** Multinomial logistic regression analysis between childhood adversity, stressful life events, traumatic life events, and latent classes of lifestyle behaviours (*n* = 1773)

		Model 1	Model 2
		Adjusted RRR (95% CI)	Adjusted RRR (95% CI)
Stressful life events	Class I (RC)	1	1
	Class II	1.73 (0.97–3.10)	1.81 (1.00–3.30)
	Class III	1.45* (1.16–1.82)	1.42* (1.12–1.81)
Childhood adversity	Class I (RC)	1	1
	Class II	1.34 (0.74–2.42)	1.30 (0.71–2.37)
	Class III	1.10 (0.90–1.36)	1.08 (0.87–1.34)
Traumatic life events	Class I (RC)	1	1
	Class II	1.35 (0.62–2.96)	1.31 (0.59–2.90)
	Class III	0.98 (0.76–1.27)	1.00 (0.77–1.30)

Class I “Poor sleep quality, low vegetable intake” (*n* = 1042), Class II “Inactive, risky alcohol use, smoker” (*n* = 58), Class III “Inactive, low fruit and vegetable intake, poor sleep quality, smoker” (*n* = 637)

Model 1 adjusted for age, sex

Model 2 adjusted for age, sex, education, income

*RRR:*Relative risk ratio, *CI* Confidence interval, *RC* Referent cluster

* *p* < 0.05

## Discussion

This study aimed to examine the associations between the experience of a range of early and later life stressful events and cluster of lifestyle behaviours in community-dwelling Australian adults. Three clusters of lifestyle behaviours were identified “Poor sleep quality, low vegetable intake” (Class I, 58.8%); “Inactive, risky alcohol use, smoker” (Class II, 3.3%); “Inactive, low fruit and vegetable intake, poor sleep quality, smoker” (Class III, 35.9%). We found that the “Inactive, low fruit and vegetable intake, poor sleep quality, smoker” cluster was more likely to have experienced stressful life events than participants in the “poor sleep quality, low vegetable intake” cluster.

We identified a cluster of protective lifestyle behaviours and two clusters of unhealthy lifestyle behaviours. Hobbs et al. [30] also found similar clusters in an Australian adult population, including a healthy cluster (physically active, non-smoker, low sitting time, consuming five servings of fruit and vegetables), unhealthy cluster (low physical activity level and consuming a poor diet) and a smaller second unhealthy cluster (alcohol abuse, long sitting time, frequent consumption of fast-food, smoking, and inadequate consumption of fruit and vegetables) [30]. In the present study, Class I characterised by having the least number of unhealthy lifestyle behaviours demonstrated clustering of multiple healthy behaviours including getting adequate physical activity, alcohol abstinence, consuming adequate fruit, being a non-smoker but with a moderate probability of meeting the vegetable intake guideline and suffering from insomnia or taking sleep medications. Our study also identified two clusters of unhealthy lifestyle behaviours with unequal distribution. Class II (3.3%) had a higher probability of being physically inactive, high risk of alcohol abuse, and being a current smoker. Class III (35.9%) had a lower probability of being physically active, consuming adequate fruit and vegetable intake, good sleep quality and being a non-smoker. This combination of unhealthy lifestyle behaviours has major overlaps with the clusters identified in a systematic review that included 37 observational studies conducted in the United Kingdom [31].

Participants in Class III who showed the most unhealthy lifestyle behaviours were more likely to have experienced stressful life events than Class I participants who showed the least unhealthy lifestyle behaviours. This finding is consistent with previous research conducted in adolescents that found a greater level of stress from life was associated with unhealthy lifestyle behaviours characterised by alcohol and substance abuse [32]. The study also found evidence that greater life stress was also associated with fewer healthy lifestyle behaviours such as consuming a healthy/weight loss diet and exercising regularly [32]. Although there is little previous research that examined the association between stressful life events and clustering of lifestyle behaviours, there is comparatively more research that examined the association between socioeconomic status and lifestyle behaviours. As low socioeconomic status is thought to reflect more stressful living environments that expose individuals to more life stressors, the evidence between socioeconomic status and lifestyle behaviours may help to illustrate the association between exposure to life stressors and lifestyle behaviours [33]. A study from a large cross-sectional survey of 27 European countries (*n* = 23,842 adults) that examined the association between socioeconomic position and clustering of lifestyle behaviours also corroborated our study findings [34]. Compared to high socioeconomic position, those individuals with the lowest socioeconomic position were more likely to belong to unhealthy behaviour clusters including being a current smoker, alcohol abuse, inadequate fruit intake, physically inactive, and infrequent use of dental check-ups [34]. In accordance with these findings, participants in Class III were less likely than their healthy counterparts to have tertiary education. Unhealthy lifestyle behaviours are known modifiable risk factors that contribute to various physical and mental health disorders [35]. Without adequate health promotion interventions, there is a greater risk of unhealthy lifestyle behaviours to accelerate the onset of chronic non-communicable diseases that requires greater dependence on extensive tertiary care within the healthcare system [36]. This evidence further reinforces the need for behavioural change interventions at the population level to reduce the current epidemic of chronic non-communicable diseases [36]. Given the rising level of psychological distress in Australia, more investment in preventive health is needed to reduce the incidence of chronic diseases [2, 36]. Moreover, psychological interventions in early ages have reduced mental health conditions in later life [37]. Hence, further investment into school-based resilience-building program such as Resourceful Adolescent Program provides intervention at an early age to effectively help adolescents develop coping strategies and regulate negative emotions from stressful situations [38]. For instance, individuals who are more resilient are less likely to demonstrate emotional eating and more likely to consume a higher quality diet [39].

One of the strengths of our study is the use of stress measures from multiple waves during 12 years of follow-up, thus providing a more comprehensive measurement. This may provide a more accurate measurement of the influence of stressors on lifestyle behaviours than a single measurement of stressors as the presence of stressors can fluctuate over time. Another strength of the present study is the use of validated tools to assess lifestyle behaviours, thus reducing the risk of bias from over- and under-reporting. Moreover, our sample is community-based to provide evidence at the broader population level rather than for specific at-risk groups.

Our study also has some limitations. Firstly, lifestyle behaviours were only assessed in the final wave. The various unhealthy lifestyle behaviours in clusters documented in our study have also been found to contribute to the development of non-communicable diseases [40], which were not incorporated in our study as these measures fall outside the scope and objectives of the present study. Thus, we did not explore these associations in this study. The narrow range of age and socioeconomic status are two sociodemographic factors that limit the generalisability of our study findings. The narrow age range of this study means that the findings are not generalisable to younger or older populations who may face other stressors. In addition, the participants living in Canberra and Queanbeyan are relatively socio-economically advantaged, which may not represent the Australian population. Despite using validated instruments to assess participants’ lifestyle behaviours, there may be inaccuracy due to misreporting that led to the misclassification of participants in categories of different lifestyle behaviours. For instance, we used a single-item measure of insomnia that could not objectively capture sleep quality, likely resulting in underestimation of the insomnia prevalence in our sample. A further limitation is the lack of dietary information from discretionary foods such as sugar-sweetened beverages and fast food given that previous research has shown that exposure to stress increases the consumption of highly palatable food [8]. A similar problem also exists with the AUDIT tool, which measures alcohol use disorders rather than consumption. Indeed, not all individuals have higher than normal alcohol consumption develop alcohol use disorder. Furthermore, there may been reduced statistical power to detect differences due to the small number of participants in the ‘Inactive, risky alcohol use, insomnia, smoker’ cluster. Workplace stressors were also of interest, but preliminary analysis did not find unique contribution and therefore future research is needed to consider the potential influence of workplace stressors on lifestyle behaviours.

## Conclusion

This study explored the prospective associations between childhood adversity, stressful life events, traumatic life events and clusters of lifestyle behaviours. Adults who experienced stressful life events were associated with the clustering of physical inactivity, low fruit and vegetable intake, and smoking. Future interventions could consider strategies for prevention of stressful life events, and/or providing appropriate support when they happen to reduce the overflow onto unhealthy behaviours, as well as behavioural change strategies that target various combinations of unhealthy lifestyle behaviours to modify the risk factors for chronic illnesses that are attributable to chronic stress.

## Supplementary Information

Supplementary Material 1.

Supplementary Material 2.

Supplementary Material 3.

## Data Availability

The datasets used can be made available from the corresponding author on reasonable request.
