# Supplementary material for: Prospective associations between stressful life course events and clusters of lifestyle behaviours
Source: BMC Public Health. 2025 Sep 24;25:3068. doi: 10.1186/s12889-025-24110-3 (PMC12461985; doi:10.1186/s12889-025-24110-3)
Supplement: Supplementary file 1 — Supplementary Material 1. [file 12889_2025_24110_MOESM1_ESM.docx]

**Survey Questionnaire**

**Childhood adversity**

Which of the following applied to your childhood? (When we say "parent" we mean "parent or parent figure").

1. I had a happy childhood
2. My parents did their best for me
3. I was neglected
4. I had a strict, authoritarian or regimented upbringing
5. I grew up in poverty or financial hardship
6. I was verbally abused by a parent
7. I suffered humiliation, ridicule, bullying or mental cruelty from a parent
8. I witnessed physical or sexual abuse of others in my family
9. I was physically abused by a parent - punched, kicked, hit or beaten with an object, or needed medical treatment
10. I received too much physical punishment - hitting, smacking etc.
11. I was sexually abused by a parent
12. Other type of mistreatment
13. I had a normal upbringing

**Stressful life events**

Have any of the following life events or problems happened to you during the last six months?

1. You yourself suffered a serious illness, injury or an assault. Yes No
2. A serious illness, injury or assault happened to a close relative. Yes No
3. Your parent, child or partner died. Yes No
4. A close family friend or another relative (aunt, cousin, grandparent) died. Yes No
5. You broke off a steady relationship. Yes No
6. You had a serious problem with a close friend, neighbour or relative. Yes No
7. You had a crisis or serious disappointment in your work or career. Yes No
8. You thought you would soon lose your job. Yes No
9. You became unemployed or you were seeking work unsuccessfully for more than one month. Yes No
10. You were sacked from your job. Yes No
11. You had a major financial crisis. Yes No
12. You had problems with the police and a court appearance. Yes No
13. Something you valued was lost or stolen. Yes No

**Traumatic life events**

Now we would like to ask you about extremely stressful or upsetting events that sometimes occur to people.

1. Did you ever have direct combat experience in a war? Yes No
2. Were you ever involved in a life-threatening accident? Yes No
3. Were you ever involved in a fire, flood or other natural disaster? Yes No
4. Did you ever witness someone badly injured or killed? Yes No
5. Were you ever raped? (that is, someone had sexual intercourse with you when you did not want to, by threatening you, or using some degree of force?) Yes No
6. Were you ever sexually molested (that is, someone touched or felt your genitals when you did not want them to)? Yes No
7. Were you ever seriously physically attacked or assaulted?Yes No
8. Have you ever been threatened with a weapon, held captive, or kidnapped? Yes No
9. Have you ever been tortured or the victim of terrorists? Yes No
10. Have you ever experienced any other extremely stressful or upsetting event? Yes No

**Physical Activity**

What do you estimate was the total time that you spent walking in this way in the LAST WEEK?

_______ Hours _______ Minutes

In the LAST WEEK, how many times did you do any vigorous gardening or heavy work around the yard, which made you breath harder or puff and pant? (Enter 0 if not at all)

__________

What do you estimate was the total time that you spent doing vigorous gardening or heavy work around the yard in the LAST WEEK?

_______ Hours _______ Minutes

The next questions exclude household chores, gardening or yard work: In the LAST WEEK, how many times did you do any vigorous physical activity which made you breathe harder or puff and pant? (eg jogging, cycling, aerobics, competitive tennis). (Enter 0 if not at all)

__________

What do you estimate was the total time that you spent doing this vigorous physical activity in the LAST WEEK?

_______ Hours _______ Minutes

In the LAST WEEK how many times did you do any other more moderate physical activities that you

have not already mentioned? (eg gentle swimming, social tennis, golf). (Enter 0 if not at all)

__________

**Sleep**

On the overall, do you think that you suffer from insomnia or sleep problems?

 Yes (1)  No (2)

In the LAST MONTH have you taken or used any pills or medications (including herbal remedies) to help you sleep?

 Yes (1)  No (2)

**Alcohol consumption**

How many standard drinks do you have on a typical day when you are drinking?

 1 or 2 (1)

 3 or 4 (2)

 5 or 6 (3)

 7 to 9 (4)

 10 or more (5)

How often do you have 5 or more standard drinks on one occasion?(for female participants)

 • Not in the last year (1)

 • Monthly or less (2)

 • 2‐3 times a month (3)

 • Once a week (4)

 • 2‐3 times a week (5)

 • 4‐6 times a week (6)

 • Every day (7)

How often do you have 7 or more standard drinks on one occasion?(for male participants)

 • Not in the last year (1)

 • Monthly or less (2)

 • 2‐3 times a month (3)

 • Once a week (4)

 • 2‐3 times a week (5)

 • 4‐6 times a week (6)

 • Every day (7)

How often during the last year have you found that you were not able to stop drinking once you had started?

 Never (1)

 Less than monthly (2)

 Monthly (3)

 Weekly (4)

 Daily or almost daily (5)

How often during the last year have you failed to do what was normally expected from you because

of your drinking?

 Never (1)

 Less than monthly (2)

 Monthly (3)

 Weekly (4)

 Daily or almost daily (5)

How often during the last year have you needed an alcoholic drink in the morning to get yourself

going after a heavy drinking session?

 Never (1)

 Less than monthly (2)

 Monthly (3)

 Weekly (4)

 Daily or almost daily (5)

How often during the last year have you had a feeling of guilt or regret after drinking?

 Never (1)

 Less than monthly (2)

 Monthly (3)

 Weekly (4)

 Daily or almost daily (5)

How often during the last year have you been unable to remember what happened the night

before because you had been drinking?

 Never (1)

 Less than monthly (2)

 Monthly (3)

 Weekly (4)

 Daily or almost daily (5)

Have you or someone else been injured as a result of your drinking?

 No (1)  Yes, but not in the last year (2)  Yes, during the last year (3)

Has a relative, friend or a doctor or other health worker been concerned about your drinking or suggested you cut down?

 No (1)  Yes, but not in the last year (2)  Yes, during the last year (3)

**Fruit and Vegetable intakes**

How many serves of vegetables do you usually eat each day?

 • 1 serve or less (1)

 • 2‐3 serves (2)

 • 4‐5 serves (3)

 • 6 serves or more (4)

 • Don’t eat vegetables (5)

How many serves of fruit do you usually eat each day?

 • 1 serve or less (1)

 • 2‐3 serves (2)

 • 4‐5 serves (3)

 • 6 serves or more (4)

 • Don’t eat fruit (5)

**Smoking**

We would now like to ask you some questions about smoking (tobacco). Do you currently smoke?

 Yes (1)  No (2)
