# Supplementary material for: Prospective associations between stressful life course events and clusters of lifestyle behaviours
Source: BMC Public Health. 2025 Sep 24;25:3068. doi: 10.1186/s12889-025-24110-3 (PMC12461985; doi:10.1186/s12889-025-24110-3)
Supplement: Supplementary file 2 — Supplementary Material 2. [file 12889_2025_24110_MOESM2_ESM.docx]

**Annotated Mplus code used in the analysis**

Title: PATH latent class analysis, 3 classes

Data: FILE = PATH 40 LCA changed coding.csv;

^a^ Variable: NAMES = id exe alcohol fruit vege insomnia smoke;

         USEVARIABLES = exe alcohol fruit vege insomnia smoke;

          Missing are all (-999);

          CATEGORICAL = exe alcohol fruit vege insomnia smoke;

          CLASSES = C(3);

^b^ Analysis: TYPE = MIXTURE;

STARTS = 5000 500;

STITERATIONS = 250;

LRTBOOTSTRAP = 500;

LRTSTARTS = 0 0 100 20;

^c^ PLOT: TYPE = PLOT3;

      SERIES = exe(1) alcohol(2) fruit(3) vege(4) insomnia(5) smoke(6);

^d^ OUTPUT: TECH10 TECH11 TECH14;

^e^ SAVEDATA: FILE = 3classes.dat;

          SAVE = CPROBABILITIES;

^a^ indicator variables in the latent class analysis to predict number of latent class with all missing values in the data file set to -999. Categorical variables and three latent classes were specified for this model.

^b^ mixture model was selected with starting value for each parameter to be estimated was specified to help with model optimisation in the iterative procedure.

^c^ plot 3 is used to create the probability plot in the manuscript with a specific order of the indicator variables in the plot.

^d^ TECH 10 was used to request univariate, bivariate, and response pattern model fit information for the categorical dependent variables in the model. TECH11 was used to request the Lo-Mendell-Rubin likelihood ratio test of model fit. TECH 14 was used to request the bootstrapped likelihood ratio test of model fit.

^e^ Output file was saved as .dat file with estimated average posterior probability of each individual and their latent class membership.

**Annotated Stata code used in the analysis**

^f^ mi set flong

mi register imputed childadv_binary cle_binary cltrauma_binary

mi register regular age_R2 gender_R2 educ

^g^ mi impute chained (logit) childadv_binary cle_binary cltrauma_binary = age_R2 i.gender_R2 i.educ, add(30) rseed(1234)

^h^ mi estimate: mlogit class i.childadv_binary i.cle_binary i.cltrauma_binary age_R2 i.gender_R2 i.educ

^i^ mi estimate, rrr

^j^ mi estimate: mlogit class i.childadv_binary i.cle_binary i.cltrauma_binary age_R2 i.educ i.gender_R2, baseoutcome(3)

mi estimate, rrr

^f^ The dataset style was set to flong in preparation for the multiple imputation to handle missing data in the dataset. Missing values in childhood adversity, stressful life events and lifetime trauma were imputed with age, gender and education variables registered as variables that are not imputed and does not contain missing values.

^g^ A multivariate imputation by chained equations was used to impute the missing values and specified the three stressors variables as binary variables and imputed using logistic regression using age, gender and education variables are predictor. 30 imputed datasets were requested to be used in the pooled analyses in the following multinomial logistic regression. Rseed sets the random seed to ensure the imputation results are replicable.

^h^ Mi estimate prefix requested estimation using the 30 imputed datasets and pool the results from the analyses. Mlogit requested a multinomial logistic regression analysis with the predicted 3 latent classes as dependent variable (categorical variable) and the independent variables of childhood adversity, stressful life events and lifetime trauma adjusted for age, gender and education level as covariates.

^i^ This command coverts the log-odds coefficients into relative risk ratios.

^j^ baseoutcome(3) change the reference category from class I to class III.
