# Supplementary material for: Prospective associations between stressful life course events and clusters of lifestyle behaviours
Source: BMC Public Health. 2025 Sep 24;25:3068. doi: 10.1186/s12889-025-24110-3 (PMC12461985; doi:10.1186/s12889-025-24110-3)
Supplement: Supplementary file 3 — Supplementary Material 3. [file 12889_2025_24110_MOESM3_ESM.docx]

Supplementary table 1. Multinomial logistic regression analysis between childhood adversity, stressful life events, traumatic life events, and latent classes of lifestyle behaviours (male, n = 787; female, n = 895).

|  |  | **Male: adjusted RRR (95% CI)** | **Female: adjusted RRR (95% CI)** |
| --- | --- | --- | --- |
| **Stressful life events** | Class I (RC) | 1 | 1 |
|  | Class II | 1.69 (0.55 – 5.18) | 1.87 (0.91 – 3.83) |
|  | Class III | **1.45* (1.04 – 2.03)** | **1.41* (1.00 – 2.00)** |
| **Childhood adversity** | Class I (RC) | 1 | 1 |
|  | Class II | 1.29 (0.42 – 3.90) | 1.24 (0.60 – 2.56) |
|  | Class III | 1.30 (0.96 – 1.76) | 0.90 (0.66 – 1.23) |
| **Traumatic life events** | Class I (RC) | 1 | 1 |
|  | Class II | 2.61 (0.33 – 20.87) | 1.09 (0.45 – 2.62) |
|  | Class III | 1.01 (0.69 – 1.49) | 0.98 (0.67 – 1.43) |

Class I “Poor sleep quality, low vegetable intake” (n=1042), Class II “Inactive, risky alcohol use, smoker” (n=58), Class III “Inactive, low fruit and vegetable intake, poor sleep quality, smoker” (n=637).

Model adjusted for age, sex, education, income.

* p<0.05

RRR: Relative risk ratio, CI: Confidence interval.
